# Supplementary material for: ESG performance and green innovation in commercial banks: Evidence from China
Source: PLoS One. 2024 Nov 7;19(11):e0308513. doi: 10.1371/journal.pone.0308513 (PMC11542845; doi:10.1371/journal.pone.0308513)
Supplement: S2 File — This document is the Stata command. (DOCX) [file pone.0308513.s002.docx]

xtreg GRDO ESG Size LEV peo ListAge Balance2 Growth i.year,fe

est store ESG

xtreg GRDO E1 Size LEV peo ListAge Balance2 Growth i.year,fe

est store E

xtreg GRDO S1 Size LEV peo ListAge Balance2 Growth i.year,fe cluster(city)

est store S

xtreg GRDO G1 Size LEV peo ListAge Balance2 Growth i.year,fe

est store G

. esttab ESG E S G using m.rtf ,replace drop(*year*) b(%7.3f) mtitles compress nogap t r2 star( * 0.1 ** 0.05 *** 0.01)

xtreg NL ESG Size LEV peo ListAge Balance2 Growth i.year,fe

est store NL

xtreg Lerner ESG Size LEV peo ListAge Balance2 Growth i.year,fe cluster(city)

est store Lerner

xtreg GRDO NL Size LEV peo ListAge Balance2 Growth i.year,fe

est store NL1

xtscc GRDO Lerner Size LEV peo ListAge Balance2 Growth i.year,fe

est store Lerner1

xtreg GRDO ESG TT1 MW Size LEV peo ListAge Balance2 Growth i.year,fe

est store MW

. esttab MW using m.rtf ,replace drop(*year*) b(%7.3f) mtitles compress nogap t r2 star( * 0.1 ** 0.05 *** 0.01)

xtreg ESG esgp Size LEV peo ListAge Balance2 Growth i.year,fe

est store one

xtivreg GRDO Size LEV peo ListAge Balance2 Growth i.year ( ESG=esgp),fe

est store two

. esttab one two using m.rtf ,replace drop(*year*) b(%7.3f) mtitles compress nogap t r2 star( * 0.1 ** 0.05 *** 0.01)

xi: xtdpd GRDO l.GRDO ESG Size LEV peo ListAge Balance2 Growth i.year, twos dgmmiv(GRDO, lag(3 3)) iv(ESG Size LEV peo ListAge Balance2 Growth i.year) lgmmiv(GRDO, lag(6))

estat abond

estat sargan

est store sys

xi: xtdpd GRDO l.GRDO ESG Size LEV peo ListAge Balance2 Growth i.year, twos dgmmiv(GRDO, lag(4 4)) iv(ESG Size LEV peo ListAge Balance2 Growth i.year)

estat sargan

estat abond

est store diff

xtreg GRDO esgp Size LEV peo ListAge Balance2 Growth i.year,fe

est store esgp

xi: xtdpd GRDO l.GRDO esgp Size LEV peo ListAge Balance2 Growth i.year, twos dgmmiv(GRDO, lag(4 4)) iv(esgp Size LEV peo ListAge Balance2 Growth i.year) lgmmiv(GRDO, lag(4))

estat abond

estat sargan

est store sys

xi: xtdpd GRDO l.GRDO esgp Size LEV peo ListAge Balance2 Growth i.year, twos dgmmiv(GRDO Balance2, lag(3 3)) iv( Size LEV peo Balance2 Growth i.year)

estat sargan

estat abond

est store diff
