# Supplementary material for: ESG performance and green innovation in commercial banks: Evidence from China
Source: PLoS One. 2024 Nov 7;19(11):e0308513. doi: 10.1371/journal.pone.0308513 (PMC11542845; doi:10.1371/journal.pone.0308513)
Supplement: Attachment — Submitted filename: Manuscript.docx [file pone.0308513.s003.docx]

**ESG performance and green innovation in commercial banks: Evidence from China**

Wang Qiliang^a,c^

Zhang Yingchun^b,c*^

Li Yang^b,c^

☆ Declarations of interest: none.

a School of Economic and Management, Qinghai Minzu University, Xining, Qinghai, P. R. China

b Business School, Qinghai University of Science and Technology, Xining, Qinghai, P. R. China

c Qinghai Provincial Key Laboratory of Big Data in Finance and Artificial Intelligence Application Technology, Xining, Qinghai, P. R. China

* Corresponding author, +86 138 9728 6366.

E-mail: [leoqlwang@icloud.com](mailto:likeqitata@icloud.com) (Wang Qiliang)

zhangych960525@163.com (Zhang Yingchun)

liyang19960909@163.com (Li Yang)

Present/permanent address: No. 3, Bayi Middle Road, Chengdong District, Xining City, Qinghai Province, 810007, P. R. China

# Abstract

Environmental, Social, and Governance (ESG) is closely related to commercial banks' promotion of "dual-carbon" goals and the concept of sustainable development. The impact of ESG performance on commercial banks' support for green innovation remains an issue for in-depth study. This paper studies 36 Chinese commercial banks in China from 2010 to 2021 and finds that the ESG performance of commercial banks can promote green innovation, and this promotion is more obvious when bank remuneration incentives are effective. Meanwhile, this paper verifies the mediating role of the non-performing loan ratio and the Lerner index in it, which provides channel support for ESG to effectively promote green innovation development. This study enriches the existing literature on environmental, social, and governance performance and green innovation in commercial banks and provides new perspectives and directions for future research.

# Keywords

ESG; green innovation; remuneration incentive; non-performing loan; Lerner index; commercial bank

# 1. Introduction

In the post-epidemic era, environmental, social, and governance problems are occurring frequently on a global scale, and "Greenhouse Earth" has become a major problem that countries around the world have to face (Green, 2023). In the modernization and development of human society in recent centuries, the development model of urbanization and industrialization to drive economic, political and social development has become the top priority for the development of the world's backward regions, but this development model will inevitably lead to further deterioration of the ecological environment. In this context, how to effectively promote social green innovation has become a key issue to be solved (DeFries et al., 2007). On this basis, commercial banks, as the core financial institutions of the indirect financial system, assume the important function of a financial reservoir in the economic system, and the green innovation of the society cannot be separated from the full support of the commercial banking system (Ding et al., 2023). Therefore, how to protect the environment while ensuring economic development and how to promote green innovation while ensuring high-quality development of commercial banks has become a key concern of scholars and practitioners around the world (Babic, 2023).

To alleviate the above-mentioned competing relationship between economic society and the ecological environment, the United Nations Environmental Programming Summer Finance Initiative (UNEP FI) put forward the concept of Environmental, Social, and Governance (ESG) in 2004, and furthermore, launched the Principles for Responsible Investment (PRI) in 2006 at the New York Stock Exchange to further implement the concept of ESG information and gradually penetrate the capital markets of countries around the world. As of June 2023, PRI has 5,372 signatories from more than 80 countries and regions, and the number is still growing. The introduction of this concept marks the beginning of a global focus on corporate social responsibility and its integration into the daily operations and long-term strategies of companies (Amini and Rahmani, 2023). On the other hand, as an important part of modernization and innovation, the study of green innovation originated in the 1990s, and its core lies in green technological innovation, in the innovation of technologies, processes, or products used to reduce environmental pollution. According to the World Intellectual Property Organization (WIPO) definition of green innovation, it includes environmentally relevant pollutant disposal and climate change mitigation-related technologies, and describes the patent classification numbers of all relevant technologies, providing a research paradigm for green innovation development (Takalo and Tooranloo, 2021).

Previous scholars have conducted a large number of studies to explore how ESG information acts on the economic system and to seek new ways of development for high-quality economic development and sustainable development of resources. Studies have confirmed that ESG disclosure has a positive impact on listed companies: specifically, ESG-advantaged firms are more likely to attract investor attention (Amel-Zadeh, 2021), and good ESG performance can significantly reduce the cost of equity capital (El et al., 2011) and the cost of debt (Goss and Roberts, 2011) and enhance corporate performance and long-term value (Friede et al., 2015), while also optimizing goodwill impairment of listed firms and improving firms' financial risk and systemic financial risk in the financial system (Nevrela, 2022). In further research, corporate innovation is found to be an important mediating mechanism for ESG to enhance corporate performance (Chen and Chang, 2013), while corporate green innovation is also an important mediating channel for digital transformation on ESG performance (Wu and Li, 2023; Zhao et al., 2023). Therefore, it can be found from previous studies that how ESG performance affects innovation, especially green innovation, has become a key issue in studying the mechanism of the role of ESG performance on the economic system.

Focusing on ESG and green innovation discussed in this paper, ESG performance is one of the effective criteria for measuring corporate sustainability performance (Rajesh, 2020), and green innovation is one of the important driving forces for low-carbon transformation and sustainable development of enterprises, and good ESG performance can have an impact on the green innovation performance of enterprises through the mechanisms of cost effect, resource effect and governance effect (Chen et al., 2006; Eccles et al., 2014). For this influence mechanism, previous researchers have broadly formed an effective theory and an ineffective theory from the perspective of risk management, market competitiveness, governance, and incentive mechanism.

Specifically, scholars of the effective theory school believe that corporate ESG performance has a positive impact on corporate green innovation (Wang et al., 2023; Zhang and Chen, 2023). First, good ESG performance can enhance firms' risk management capabilities and reduce the risk of violating environmental regulations and potential negative impacts, thus releasing more resources to be invested in green innovation (Clarkson et al., 2008; Darnall et al., 2008; Khan et al., 2016). Second, ESG advantages can enhance firms' market competitiveness and promote green innovation as parties pay more attention to sustainability issues (Chen, Y. S., 2006; Schaltegger and Wagner, 2011). Again, good ESG performance usually represents efficient corporate governance, including transparent decision-making processes, effective monitoring mechanisms, and fair distribution of rights and benefits, which in turn facilitates the flow of information within the firm and thus promotes the implementation of green innovation (Dangelico and Pujari, 2010; Chen et al., 2012; Triguero et al., 2013). Finally, incorporating ESG objectives into incentive mechanisms, such as performance appraisal indicators for management, can effectively encourage management and employees to participate in and promote green innovation (Eccles and Serafeim,2013; Amel-Zadeh and Serafeim,2018). In addition, scholars have scaled up the above facilitation effects to the national level to study the macro effects (Long et al., 2023), or focused on large-scale manufacturing and industrial enterprises to study the micro-mechanisms (Rehman et al., 2021; Zhu et al., 2023).

In contrast, scholars of the nullist school argue that there is a time-lag effect in the belief that corporate ESG performance has an impact on green innovation, and that green innovation cannot be released in a timely and effective manner by ESG information (Eccles et al., 2014), and that excessive attention to ESG by listed firms can divert the attention and resources of the firms, and can even hurt green innovation (Servaes and Tamayo, 2013; Luo et al., 2015). To summarize, the study of the relationship between ESG information and green innovation has become one of the key topics of in-depth drilling by various research units. However, the existing research literature pays more attention to non-financial institutions, and the research results on the relationship between ESG and green innovation of commercial banks are slightly thin, which to a certain extent is not conducive to the development of high-quality paths of the economy from the financial system of indirect finance. Therefore, this paper tries to grasp the special subject of commercial banks to conduct targeted research on the influence mechanism of the above two.

Based on the above research background, this paper will draw on academic research on non-financial institutions to examine the impact of commercial banks' ESG performance on green innovation from the perspectives of risk management, market competitiveness, governance, and incentives. The possible marginal contributions of this paper are as follows: first, research perspective. Current academic research on banks' innovation drivers is saturated, but little literature has explored the social responsibility perspective. This paper explores the impact of banks' ESG performance on green innovation, providing a new theoretical perspective for academic research in the field of bank innovation. Second, the mechanism. This paper investigates the role of banks' ESG responsibilities, and discusses the impact of banks' ESG performance on green innovation from the perspectives of management compensation (governance and incentive mechanism), non-performing loan ratio (representing risk management), and Lerner's index (reflecting market competitiveness). This theoretical result provides new insights for academic research in this area and fills the previous research gap. Third, policy support. Studying the impact of banks' ESG performance on green innovation helps policymakers to incorporate ESG factors in the regulatory framework, thus guiding the banking industry towards sustainable development.

The remainder of the paper is organized as follows. Section “Theory and hypotheses” presents theory introduction and hypothesis development. Section “Data and methodology” goes over the data sources, variable definitions, and model. Section “Empirical results” presents the descriptive statistics and baseline empirical findings. Section “Robustness test” presents endogeneity test and robustness test results. Section “Further analysis” presents heterogeneity analysis and further analysis results. Lastly, the section “Conclusion and policy implications” concludes and gives policy implications.

# 2. Theory and hypotheses

*2.1.ESG performance and green innovation*

ESG performance will make other participants in the economy and society pay attention to the ESG behavior of commercial banks, and take the social responsibility performance of commercial banks as one of the decision-making factors, which creates a new behavioral constraint on commercial banks, and then positively incentivizes the green innovation of commercial banks.

On the one hand, based on the information asymmetry theory, green innovation activities require a long return cycle and the return benefit is not clear, and the problems of adverse selection and moral hazard are particularly serious (Arena et al., 2015); therefore, if there are high information barriers inside and outside of the commercial bank, the negative impact on the green innovation activities will be huge (Chen et al., 2018). And when commercial banks have better ESG rating performance, externally, external investors can intuitively feel the contribution of commercial banks to society in the field of green environmental protection through ESG ratings (Cao et al., 2022), which can better promote the formation of a tripartite partnership between the enterprise, the market, and the government, and push commercial banks to further support green R&D investment (Feng and Ma, 2023); from an internal perspective, commercial banks can judge the necessity of green innovation project investment through ESG rating information, reduce the threshold of brown industry's investment in green innovation, and effectively alleviate the innovation dilemma of information asymmetry (Hu et al., 2023). In summary, the ESG performance of commercial banks can alleviate the problem of internal and external information barriers, and by improving the information environment, it can then promote the efficiency of green innovation in commercial banks.

On the other hand, based on the theory of limited attention, economic individuals have limited cognitive abilities (Kahneman, 1973) and need to selectively allocate their attention to different things (Xu et al., 2023), so investors are more inclined to pay attention to macro-market information while relatively neglecting micro-firm information (Peng and Xiong, 2006), and identify firms' information partially to third-party organizations. When this cognitive fairness does not exist, economic individuals may not even be able to effectively recognize the information at all (Kahneman, 2003). At a time of increasing concern about the social responsibility of commercial banks, the ESG performance of commercial banks given by independent rating agencies can inform a wide range of economic individuals of the efficiency of a bank's green responsibility. This leads to further capital pooling and supports green innovation of commercial banks, creating positive feedback.

In summary, we suppose that:

**Hypothesis 1.** Commercial banks' ESG performance has a facilitating effect on their green innovation.

*2.2. The moderating effect of remuneration incentives*

The compensation incentive system implies that if the firm performs well, the management will benefit at the same time, solving the credit risk and principal-agent problem of the management by aligning their interests with those of the firm (Chen et al., 2006). Established studies have demonstrated that compensation incentives can effectively promote corporate innovation (Biggerstaff et al., 2019; Zhou et al., 2021); thus, commercial banks can positively regulate the support of green innovation from commercial banks' ESG performance by incorporating ESG performance into management compensation assessment indicators (Zhao et al., 2023). 2023). Therefore, we suppose that:

**Hypothesis 2.** When commercial banks have higher remuneration incentives, the positive correlation between ESG performance on green innovation is higher.

*2.3. The mediating effect of non-performing loan (NPL)*

One of the central features of the banking system and the financial crisis is ex-post credit risk in the form of non-performing loans (Anastasiou, 2023; Louzis et al., 2012), where credit expansion during periods of economic growth is exposed during periods of economic adjustment, which in turn creates non-performing loans (Manz, 2019; Foos et al., 2010), exposing banks exposed to significant credit risk, creating structural financial risk to the economy and society (Ghosh, 2017). In contrast, banks with good ESG performance care about the interests of their stakeholders and therefore carefully screen borrowers and monitor loan utilization to reduce the risk of default, which in turn reduces the NPL ratio (Liu et al., 2023; Chiaramonte et al., 2022; Citterio and King, 2023). At the same time, the decline in the NPL ratio will enhance the investment confidence of banks, making them more willing to expand the scale of lending, which in turn will synchronize the increase in the volume of loans invested in the green sector (Zhang, B, 2011), eliminating the negative effects of financial barriers on green innovation (Ghisetti et al., 2017), and facilitating green innovation in the economy and society. Therefore, we suppose that:

**Hypothesis 3.** NPL ratios play a mediating role in commercial banks' ESG performance affecting green innovation.

*2.4. The mediating effect of the Lerner Index*

The Lerner Index is used to measure the degree of market competition of firms in an industry (Lerner, 1934; Elzinga et al., 2011). Established studies have found that commercial banks' top-performing ESG indicators can attract investors' attention and capital inflows, which can drive up the share price, which in turn increases the bank's Lerner Index and enhances its competitive advantage (Rabaya et al., 2022; Aboud et al., 2018; Aouadi and Marsat, 2018). Meanwhile, the Lerner index has a significant contributing role in promoting green innovation. Monopolistic commercial banks with high Lerner indexes usually have more resources and capital (Pankratov and Trifonov, 2021), and can provide large and targeted green financial products to meet the financial needs of green innovation (Silva and Carreira, 2012; Kim et al., 2021), enabling monopolistic banks to better support and invest in green innovation projects (Xia and Liu, 2022), can focus more opportunities on sustainable development and green economy, and provide a solid economic foundation for promoting green innovation. Therefore, we suppose that:

**Hypothesis 4.** The Lerner Index plays a mediating role in the process by which commercial banks' ESG performance affects green innovation.

**Figure 1.**

Theoretical framework diagram.

# 3. Research design

*3.1. Date*

As of 2022, there are a total of 41 bank stocks listed on China's A-share market, specifically including 19 joint-stock commercial banks, 4 policy banks, 4 city commercial banks, 9 agricultural and commercial banks, and 5 village banks. Excluding the banks in which key data are missing, a total of 36 commercial banks obtained valid data. Therefore, this paper selects 2010 to 2021 as the research sample to analyze the impact of ESG performance on green innovation development in China's banking industry. The data used are from the WIND database and the CNRDS database, and some missing data are filled in manually by checking the annual reports of commercial banks.

*3.2. Variable definition*

*3.2.1 ESG performance (ESG)*

Referring to Yin et al. (2023), we selected the Sino-Securities Index (SNSI) ESG Scores published by ChinDices as the core explanatory variable, and three separate scores for environment (*E1*), society (*S1*), and governance (*G1*) were selected to test the impact of different dimensions on green innovation respectively. Meanwhile, ChinDices publishes ESG Ratings together (9 grades from C to AAA), we assign separate scores to them, and use the results as instrumental variables for endogeneity testing.

*3.2.2 Green Innovation (GRDO)*

The current academic research on enterprise technology innovation measurement indexes focuses on R&D inputs, patent outputs, and R&D personnel ratios (Hagedoorn and Cloodt, 2003), which is still applicable in the study of green innovation. Compared with R&D inputs and R&D personnel ratios, patent outputs are more representative of the degree of development of corporate science and technology innovation (Xie et al., 2019; Singh et al., 2020). Therefore, this paper selects the annual number of green inventions independently filed (*GRDO*) as a proxy variable for green innovation, while the number is treated as a natural logarithm to ensure the smoothness of the data, which is obtained from the CNRDS database.

*3.2.3 Remuneration Incentives (MW)*

Referring to the studies of Conyon and He (2012) and Cui et al. (2021), this paper chooses the compensation received by the company's senior management based on their position and performance, i.e., the annual management wealth (*MW*), as a proxy variable for remuneration incentives; meanwhile, this quantity is treated as a natural logarithm to ensure the smoothness of the data, which is obtained from the WIND database.

*3.2.4 Non-performing Loan (NPL)*

Referring to Kryzanowski et al. (2023), the annual non-performing loan ratio (*NPL*), defined as non-performing loans divided by total loans, was selected as one of the mediating variables from the WIND database.

*3.2.5 Lerner Index (Lerner)*

Referring to Angelini and Cetorelli (2003), the degree of competition of commercial banks is measured using the Lerner index (*Lerner*) based on individual bank data from the WIND database.

*3.2.6 Control variables*

To further improve the explanatory degree of the regression model, drawing on Dietrich and Wanzenried (2011), total bank asset size, gearing ratio, total number of employees, number of years the firm has been in existence, equity checks and balances, and operating growth rate are selected as control variables to be added to the regression, and the above data are obtained from the WIND database. The specific selection of indicators are shown in Table 1.

**Table 1**

Variable definitions.

| **Type** | **Variable** | **Measurement** |
| --- | --- | --- |
| Dependent variable | GRDO | ln (Annual number of green inventions independently filed+1) |
| Independent variable | ESG | ESG rating scores |
|  | E1 | Environmental dimension rating scores |
|  | S1 | Social dimension rating scores |
|  | G1 | Governance dimension rating scores |
| Moderating variable | *MW* | ln (Annual remuneration of management+1) |
| Mediating variable | *NPL* | non-performing loans divided by total loans |
|  | *Lerner* | Lerner index |
| Control variabes | Size | Total asset |
|  | LEV | Asset-Liability ratio |
|  | peo | ln (Total number of employees+1) |
|  | ListAge | Years of business establishment |
|  | Balance | Shareholding checks and balances |
|  | Growth | Revenue growth rate |

*3.3. Model setting*

We construct the following model:

$$\mathrm{GRDO}_{it}=\alpha+\beta_{1}\mathrm{ESG}_{it}+\beta_{2}\mathrm{Size}_{it}+\beta_{3}\mathrm{LEV}_{it}+\beta_{4}\mathrm{peo}_{it}+\beta_{5}\mathrm{ListAge}_{it}+\beta_{6}\mathrm{Balance}_{it}+\beta_{7}\mathrm{Growth}_{it}+{bank}_{i}+{time}_{t}+\varepsilon_{it}$$

Where $\mathrm{GRDO}_{it}$ is the explanatory variable, indicating green innovation; $\mathrm{ESG}_{it}$ is the core explanatory variable, indicating ESG performance; $\mathrm{Size}_{it}$, $\mathrm{LEV}_{it}$, $\mathrm{peo}_{it}$, $\mathrm{ListAge}_{it}$, $\mathrm{Balance}_{it}$, $\mathrm{Growth}_{it}$is the control variable. We also control for the individual fixed effect ${bank}_{i}$ and time fixed effect ${time}_{t}$.

In this model, this study focuses on the coefficient $\beta_{1}$ of influence of the core explanatory variables $\mathrm{ESG}_{it}$ on the explanatory variables $\mathrm{GRDO}_{it}$. If the coefficient $\beta_{1}$ is significantly positive, it represents that the ESG performance of Chinese commercial banks can significantly promote green innovation; on the contrary, if the coefficient $\beta_{1}$ is significantly negative, it represents that the ESG performance of Chinese commercial banks can significantly inhibit green innovation. According to the previous theoretical analysis, this paper expects the coefficient $\beta_{1}$ to be significantly positive.

Further, in this paper, in order to explore the moderating effect of management compensation as a moderating variable on the benchmark effect, the model for the mechanism analysis is set up as follows:

$${GRDO}_{it}=\alpha+\beta_{1}{ESG\times MW}_{it}+\beta_{2}{ESG}_{it}+\beta_{3}{MW}_{it}+\beta_{4}{Size}_{it}+\beta_{5}{LEV}_{it}+\beta_{6}{peo}_{it}+\beta_{7}{ListAge}_{it}+\beta_{8}{Balance}_{it}+\beta_{9}{Growth}_{it}+{bank}_{i}+{time}_{t}+\varepsilon_{it}$$

In this model, $\mathrm{MW}_{it}$ is the moderator variable, which represents the annual management compensation of commercial banks; ${\mathrm{MW}\times\mathrm{ESG}}_{it}$ is the core explanatory variable of the moderating effect, which represents the cross-multiplication of the treatment variable and the moderating variable.

In this model, this study focuses on the influence coefficient $\beta_{1}$of ${\mathrm{ESG}\times\mathrm{MW}}_{it}$, the cross-multiplier of the treatment variable and the moderator variable, if the coefficient is significantly positive, it represents the positive moderating benchmark effect of the management compensation; on the contrary, if the coefficient is significantly negative, it represents the negative moderating benchmark effect of the management compensation. According to the previous theoretical analysis, this paper expects the coefficient $\beta_{1}$ is significantly positive.

In addition, in order to explore the role channels of benchmark effect, this paper analyzes two different role channels of non-performing loan ratio and market competitiveness, and the model setting for the analysis of the above channels is as follows:

$${NPL}_{it}=\gamma+\gamma_{1}\mathrm{ESG}_{it}+\gamma_{2}\mathrm{Size}_{it}+\gamma_{3}\mathrm{LEV}_{it}+\gamma_{4}\mathrm{peo}_{it}+\gamma_{5}\mathrm{ListAge}_{it}+\gamma_{6}\mathrm{Balance}_{it}+\gamma_{7}\mathrm{Growth}_{it}+{bank}_{i}+{time}_{t}+\varepsilon_{it}$$

$$\mathrm{GRDO}_{it}=\alpha+\beta_{1}{NPL}_{it}+\beta_{2}\mathrm{Size}_{it}+\beta_{3}\mathrm{LEV}_{it}+\beta_{4}\mathrm{peo}_{it}+\beta_{5}\mathrm{ListAge}_{it}+\beta_{6}\mathrm{Balance}_{it}+\beta_{7}\mathrm{Growth}_{it}+{bank}_{i}+{time}_{t}+\varepsilon_{it}$$

$$\mathrm{Lerner}_{it}=\gamma+\gamma_{1}\mathrm{ESG}_{it}+\gamma_{2}\mathrm{Size}_{it}+\gamma_{3}\mathrm{LEV}_{it}+\gamma_{4}\mathrm{peo}_{it}+\gamma_{5}\mathrm{ListAge}_{it}+\gamma_{6}\mathrm{Balance}_{it}+\gamma_{7}\mathrm{Growth}_{it}+{bank}_{i}+{time}_{t}+\varepsilon_{it}$$

$$\mathrm{GRDO}_{it}=\alpha+\beta_{1}\mathrm{Lerner}_{it}+\beta_{2}\mathrm{Size}_{it}+\beta_{3}\mathrm{LEV}_{it}+\beta_{4}\mathrm{peo}_{it}+\beta_{5}\mathrm{ListAge}_{it}+\beta_{6}\mathrm{Balance}_{it}+\beta_{7}\mathrm{Growth}_{it}+{bank}_{i}+{time}_{t}+\varepsilon_{it}$$

Where ${NPL}_{it}$ is the mediating variable, which represents the non-performing loan ratio of commercial banks; $\mathrm{Lerner}_{it}$ is another mediating variable and represents the Lerner index of commercial banks.

In the mediated effects model of the NPL ratio, this study focuses on the coefficient $\gamma_{1}$ of the effect of the treatment variable $\mathrm{ESG}_{it}$ on the mediating variable ${NPL}_{it}$, and the coefficient $\beta_{1}$ of the effect of the mediating variable ${NPL}_{it}$ on the outcome variable $\mathrm{GRDO}_{it}$. Based on the theoretical analysis in the previous section, this paper expects the coefficients $\gamma_{1}$ to be significantly negative and $\beta_{1}$ to be significantly negative.

In the mediation effect model of market competition, this study focuses on the coefficient $\gamma_{1}$ of the treatment variable $\mathrm{ESG}_{it}$ on the mediator variable $\mathrm{Lerner}_{it}$, and the coefficient $\beta_{1}$ of the mediator variable $\mathrm{Lerner}_{it}$ on the outcome variable $\mathrm{GRDO}_{it}$. Based on the theoretical analysis in the previous section, this paper expects that the coefficients $\gamma_{1}$ are significantly positive and $\beta_{1}$ are significantly positive.

# 4. Empirical results

*4.1. Descriptive statistics*

This paper provides descriptive statistics for each variable. From the statistical results of 263 observations, the mean value of Esg rating score is 80.129, the minimum value is 65.3, and the maximum value is 87.98, which indicates that the rating index of China's commercial banking industry is basically at the medium to high level. The statistical index characteristics of the remaining data are shown in Table 2.

**Table 2**

Descriptive statistics.

| **Variable** | **N** | **Mean** | **SD** | **Min** | **Max** |
| --- | --- | --- | --- | --- | --- |
| GRDO | 263 | 0.92 | 1.458 | 0 | 6.48 |
| ESG | 263 | 80.129 | 3.903 | 65.3 | 87.98 |
| E1 | 263 | 70.009 | 5.81 | 56.3 | 88.279 |
| S1 | 263 | 77.847 | 5.616 | 60.59 | 89.49 |
| G1 | 263 | 86.064 | 5.891 | 56.45 | 96.52 |
| MW | 263 | 7.616 | 0.601 | 4.796 | 9.053 |
| NL | 263 | 1.284 | 0.406 | 0.38 | 2.47 |
| Lerner | 263 | 0.356 | 0.157 | 0.002 | 0.683 |
| Size | 263 | 10.101 | 1.511 | 6.94 | 12.771 |
| LEV | 263 | 92.877 | 1.124 | 89.667 | 96.588 |
| peo | 263 | 10.316 | 1.631 | 7.21 | 13.129 |
| ListAge | 263 | 1.926 | 0.839 | 0 | 3.434 |
| Balance2 | 263 | 1.529 | 0.76 | 0.241 | 3.845 |
| Growth | 263 | 0.135 | 0.132 | -0.21 | 0.542 |

*4.2. The main empirical results*

Table 3 shows the regression results of commercial banks' ESG performance on green innovation, in which the regression results are categorized into four groups to verify the coefficients of the impact of the full dimension, environmental dimension, social dimension and governance dimension on green innovation. In column (1), the regression coefficient of ESG overall evaluation score is 0.056, which is significant at 1% level,indicating that ESG performance of commercial banks can significantly contribute to the development of green innovation. In columns (2), (3), and (4), the regression coefficient of the environmental dimension is 0.03, which is significant at the 5% level; the regression coefficient of the social dimension is 0.019, which is significant at the 5% level; and the regression coefficient of the corporate governance dimension is 0.038, which is significant at the 1% level, and it can likewise indicate that the ESG performance of the commercial banks can contribute to the development of green innovation.

**Table 3**

Baseline results.

| **VARIABLES** | *GRDO* | | | |
| --- | --- | --- | --- | --- |
|  | (1) | (2) | (3) | (4) |
| *ESG* | 0.056^***^ | - | - | - |
|  | (2.95) | - | - | - |
| *E1* | - | 0.030^**^ | - | - |
|  | - | (2.26) | - | - |
| *S1* | - | - | 0.019^**^ | - |
|  | - | - | (2.75) | - |
| *G1* | - | - | - | 0.038^***^ |
|  | - | - | - | (2.77) |
| *Size* | -1.276 | -1.383 | -1.324 | -1.413 |
|  | (-1.29) | (-1.38) | (-0.84) | (-1.42) |
| *LEV* | 0.250^**^ | 0.270^**^ | 0.247 | 0.256^**^ |
|  | (2.17) | (2.32) | (1.40) | (2.22) |
| *peo* | -2.027^***^ | -1.868^***^ | -2.015^**^ | -1.935^***^ |
|  | (-3.36) | (-3.08) | (-2.58) | (-3.21) |
| *ListAge* | -0.695^***^ | -0.650^***^ | -0.671^**^ | -0.679^***^ |
|  | (-3.31) | (-3.07) | (-2.13) | (-3.23) |
| *Balance2* | -0.549^**^ | -0.574^**^ | -0.610^***^ | -0.570^**^ |
|  | (-2.26) | (-2.34) | (-2.99) | (-2.34) |
| *Growth* | 0.355 | 0.419 | 0.285 | 0.189 |
|  | (0.59) | (0.69) | (0.42) | (0.31) |
| *Constant* | 5.028 | 5.072 | 8.797 | 6.002 |
|  | (0.55) | (0.55) | (0.97) | (0.66) |
| Observations | 263 | 263 | 263 | 263 |
| R-squared | 0.546 | 0.539 | 0.533 | 0.544 |
| *Year* | Control | Control | Control | Control |
| *Bank* | Control | Control | Control | Control |

Note: Robust standard errors are reported in parenthesis. *, **, and *** denote 10%, 5%, and 1% significance levels, respectively.

# 5. Robustness test

*5.1.* *Reverse causality issues*

This paper verifies the reverse causality bias that while ESG affects green innovation, green innovation in commercial banks itself, as a socially responsible behavior, likewise improves banks' ESG performance. Referring to Yin et al. (2023), a two-stage least squares (2SLS) regression was conducted as an instrumental variable after assigning ESG ratings. From the regression results, in the first stage, the instrumental variables of ESG ratings show a significant facilitating effect on ESG ratings, and in the second stage, the instrumental variables still have a significant facilitating effect on green innovation in commercial banks, proving that the endogeneity test is passed.

**Table 4**

2SLS regression: reverse causality issues.

| **VARIABLES** | (1) | (2) |
| --- | --- | --- |
|  | first stage | second stage |
|  | *ESG* | *GRDO* |
| *ESG* | - | 0.045^**^ |
|  | - | (2.13) |
| *ESG_Rating* | 3.970^***^ | - |
|  | (28.20) | - |
| *Size* | 1.197 | -1.314 |
|  | (0.72) | (-1.32) |
| *LEV* | -0.028 | 0.253^**^ |
|  | (-0.14) | (2.19) |
| *peo* | 0.172 | -2.002^***^ |
|  | (0.17) | (-3.32) |
| *ListAge* | 0.261 | -0.689^***^ |
|  | (0.75) | (-3.28) |
| *Balance2* | -0.929^**^ | -0.563^**^ |
|  | (-2.31) | (-2.31) |
| *Growth* | 1.350 | 0.336 |
|  | (1.34) | (0.56) |
| *Constant* | 48.477^***^ | 5.753 |
|  | (3.23) | (0.63) |
| Observations | 263 | 263 |
| R-squared | 0.890 | 0.528 |
| *Year* | Control | Control |
| *Bank* | Control | Control |

Note: Robust standard errors are reported in parenthesis. *, **, and *** denote 10%, 5%, and 1% significance levels, respectively.

*5.2. Dynamic panel issues*

This paper also uses System Generalized Method of Moments (SYS-GMM) and Difference Generalized Method of Moments (DIF-GMM) to test the dynamic panel bias, i.e., past green innovations may affect future green innovations, and Table 5 shows the regression results. The regression coefficients of commercial banks' ESG performance in the two columns are 0.014 and 0.026, respectively, and are significant at the 1% level and 10% level, respectively, proving that the endogeneity test is passed.

**Table 5**

The GMM method: dynamic panel issues.

| **VARIABLES** | *GRDO* | |
| --- | --- | --- |
|  | (1) | (2) |
|  | SYS-GMM | DIF-GMM |
| *ESG* | 0.014^***^ | 0.026^*^ |
|  | (2.68) | (1.96) |
| *L.GRDO* | 0.572^***^ | 0.198^*^ |
|  | (6.69) | (1.80) |
| *Size* | -0.069 | -0.186 |
|  | (-0.77) | (-1.30) |
| *LEV* | -0.140^***^ | -0.224^***^ |
|  | (-2.68) | (-2.75) |
| *peo* | 0.339^***^ | 0.546^***^ |
|  | (3.35) | (3.35) |
| *ListAge* | 0.019 | 0.074 |
|  | (0.48) | (1.05) |
| *Balance2* | -0.066 | -0.153^*^ |
|  | (-1.21) | (-1.90) |
| *Growth* | -0.350 | -0.325 |
|  | (-1.13) | (-1.03) |
| *Constant* | 9.630^**^ | 15.781^**^ |
|  | (2.09) | (2.11) |
| Observations | 224 | 224 |
| *AR（1）* | 0.018 | 0.041 |
| *AR（2）* | 0.685 | 0.962 |
| *sargan* | 0.744 | 0.394 |
| *Year* | Control | Control |
| *Bank* | Control | Control |

Note: Robust standard errors are reported in parenthesis. *, **, and *** denote 10%, 5%, and 1% significance levels, respectively.

*5.3. Replacement of explanatory variables*

Finally, in order to further reflect the robustness of the regression results of this study, we also include ESG rating as a proxy variable for the robustness test. As can be seen from the regression results in Table 6, the regression coefficients of ESG rating grade,are all positive and significant at the 5% level, indicating that the proxy variables can still have a promotional effect on the development of green innovation in commercial banks, and the robustness test is passed.

**Table 6**

Replacement of explanatory variables.

| **VARIABLES** | *GRDO* | | |
| --- | --- | --- | --- |
|  | (1) | (2) | (3) |
|  | LSDV | SYS-GMM | DIF-GMM |
| *ESG* | 0.180^**^ | 0.076^**^ | 0.221^**^ |
|  | (2.11) | (1.96) | (2.36) |
| *L.GRDO* | - | 0.303^**^ | 0.776^***^ |
|  | - | (2.07) | (12.67) |
| *Size* | -1.260 | -0.161 | 0.265 |
|  | (-1.25) | (-1.24) | (1.60) |
| *LEV* | 0.252^**^ | -0.212^***^ | -0.175^***^ |
|  | (2.16) | (-2.62) | (-2.65) |
| *peo* | -1.994^***^ | 0.481^***^ | 0.001 |
|  | (-3.28) | (2.78) | (0.01) |
| *ListAge* | -0.677^***^ | 0.033 | -0.143 |
|  | (-3.19) | (0.51) | (-0.74) |
| *Balance2* | -0.605^**^ | -0.168^**^ | 0.042 |
|  | (-2.48) | (-2.21) | (0.60) |
| *Growth* | 0.397 | -0.172 | -0.114 |
|  | (0.65) | (-0.46) | (-0.30) |
| *Constant* | 7.952 | 16.794^**^ | 13.057^**^ |
|  | (0.87) | (2.27) | (2.20) |
| Observations | 263 | 224 | 224 |
| R-squared | 0.537 | - | - |
| *AR（1）* | - | 0.074 | 0.024 |
| *AR（2）* | - | 0.944 | 0.677 |
| *sargan* | - | 0.817 | 0.816 |
| *Year* | Control | Control | Control |
| *Bank* | Control | Control | Control |

Note: Robust standard errors are reported in parenthesis. *, **, and *** denote 10%, 5%, and 1% significance levels, respectively.

# 6. Further analysis

*6.1. Moderating mechanism analysis*

Table 7 shows the test of the moderating effect of management compensation. In this regression result, we only need to observe the regression result of the cross-multiplier term between commercial banks' ESG performance and management's annual compensation.The regression coefficient of *ESG×MW* is 4.211, which is significant at the 10% level, indicating that management's compensation enhancement can positively modulate the promotional effect of commercial banks' ESG performance on the development of green innovation.

**Table 7**

Moderating mechanism analysis.

| **VARIABLES** | *GDRO* |
| --- | --- |
|  | (1) |
| *ESG×MW* | 4.211^*^ |
|  | (1.77) |
| *ESG* | 0.051^***^ |
|  | (2.64) |
| *MW* | -0.033 |
|  | (-0.22) |
| *Size* | -1.183 |
|  | (-1.19) |
| *LEV* | 0.264^**^ |
|  | (2.28) |
| *peo* | -2.061^***^ |
|  | (-3.43) |
| *ListAge* | -0.739^***^ |
|  | (-3.48) |
| *Balance2* | -0.535^**^ |
|  | (-2.19) |
| *Growth* | 0.186 |
|  | (0.31) |
| *Constant* | 3.954 |
|  | (0.43) |
| Observations | 263 |
| R-squared | 0.553 |
| *Year* | Control |
| *Bank* | Control |

Note: Robust standard errors are reported in parenthesis. *, **, and *** denote 10%, 5%, and 1% significance levels, respectively.

*6.2. Mediating mechanism analysis*

Referring to MacKinnon (2021), Table 8 shows the mediation effect test for NPL ratio and Lerner index. In particular, the (1) and (2) columns show the regression results of commercial banks' ESG performance on NPL ratio and Lerner index; the (3) and (4) columns show the regression results of NPL ratio and Lerner index on green innovation.

On the one hand, it can be seen from the regression results of ESG performance of commercial banks on NPL ratio in the (1) column. The regression coefficient of commercial banks' ESG performance is -0.016, which is significant at the 1% level, indicating that with the improvement of commercial banks' ESG performance can significantly suppress the NPL ratio. Combined with the regression results of NPL ratio on green innovation in the (3) column, it can be seen that the regression coefficient of NPL ratio is -0.666, which is significant at the 5% level, indicating that the NPL ratio can inhibit the development of green innovation in commercial banks. To summarize, commercial banks' ESG performance can enhance the development of green innovation by suppressing the NPL ratio.

On the other hand, the regression result of commercial banks' ESG performance on Lerner index in the (2) column shows that the regression coefficient of commercial banks' ESG performance is 0.001, which is significant at the 10% level, indicating that commercial banks' ESG performance can have a promoting effect on the improvement of Lerner index. Also in the (4) column, the regression coefficient of Lerner index on green innovation of commercial banks is 2.503, which is significant at the 5% level, indicating that Lerner index has a significant promotion effect on green innovation. To summarize, commercial bank ESG performance has a facilitating effect on green innovation development by enhancing the competitiveness of commercial banks.

**Table 8**

Mediating mechanism analysis.

| **VARIABLES** | (1) | (2) | (3) | (4) |
| --- | --- | --- | --- | --- |
|  | *NPL* | *Lerner* | *GRDO* | *GRDO* |
| *ESG* | -0.016^***^ | 0.001^*^ | - | - |
|  | (-3.77) | (1.89) | - | - |
| *NL* | - | - | -0.666^**^ | - |
|  | - | - | (-2.27) | - |
| *Lerner* | - | - | - | 2.503^**^ |
|  | - | - | - | (2.70) |
| *Size* | -0.095 | -0.200^**^ | -1.501 | -0.965 |
|  | (-0.42) | (-2.86) | (-1.50) | (-1.54) |
| *LEV* | -0.060^**^ | 0.002 | 0.222^*^ | 0.259^***^ |
|  | (-2.25) | (0.24) | (1.88) | (3.38) |
| *peo* | -0.040 | 0.105^***^ | -1.946^***^ | -2.165^***^ |
|  | (-0.29) | (2.97) | (-3.21) | (-6.29) |
| *ListAge* | -0.218^***^ | 0.037^**^ | -0.812^***^ | -0.754^***^ |
|  | (-4.52) | (2.29) | (-3.66) | (-3.21) |
| *Balance2* | 0.299^***^ | -0.031^**^ | -0.412 | -0.544^***^ |
|  | (5.35) | (-2.16) | (-1.58) | (-3.59) |
| *Growth* | -0.554^***^ | -0.027 | -0.095 | 0.329 |
|  | (-4.02) | (-1.10) | (-0.15) | (1.03) |
| *Constant* | 9.110^***^ | 1.015 | 14.186 | 6.101 |
|  | (4.37) | (1.42) | (1.51) | (1.56) |
| Observations | 263 | 263 | 263 | 263 |
| R-squared | 0.765 | 0.801 | 0.539 | - |
| *Year* | Control | Control | Control | Control |
| *Bank* | Control | Control | Control | Control |

Note: Robust standard errors are reported in parenthesis. *, **, and *** denote 10%, 5%, and 1% significance levels, respectively.

Note

# 7. Conclusion

After studying the influence mechanism of ESG on green innovation of commercial banks, this paper draws the following conclusions:

First, ESG has a facilitating effect on the green innovation of commercial banks, and the conclusion is robust to 2SLS and GMM tests for reverse causality and dynamic panel bias. The findings of the study provide useful insights for policymakers and bank management: by enhancing the ESG performance of commercial banks, the development of green innovation can be effectively promoted to realize the goal of sustainable development.

Second, management compensation incentives can positively moderate the causal relationship between ESG performance and green innovation in commercial banks. To further expand this moderating effect, commercial banks can further improve the compensation incentive mechanism for management, incorporate ESG performance into the compensation incentive assessment criteria, and incentivize management to play a positive role in green innovation by giving them incentives related to ESG objectives, thereby promoting the development of green innovation in commercial banks.

Thirdly, this paper investigates the mediating effects of the NPL ratio and Lerner's index in the baseline effect, where commercial banks' ESG performance promotes green innovation by lowering the NPL ratio and increasing the Lerner's index. The results of this study suggest that government departments can promote green innovation by improving the ESG mandatory disclosure system, nature protection laws, and other laws and regulations, and by improving the transmission efficiency among explanatory, mediating, and interpreted variables.

Based on this, the paper makes the following policy recommendations: First, banks need to set clear ESG objectives and integrate them into their strategic planning and performance assessment systems. This will help to raise management's awareness of ESG issues and ensure that ESG objectives are effectively implemented, thereby promoting green innovation in banks and further addressing the "dual carbon" issue. Second, commercial banks can establish a comprehensive environmental risk management framework, including improving management compensation incentives, environmental due diligence, and sustainable investment. By incorporating environmental risks into management compensation incentives, banks can direct funds to projects and enterprises that are in line with the development of green innovation, thus further promoting incentives for green innovation. Third, commercial banks should actively cooperate and communicate with stakeholders such as the government, regulatory agencies, environmental organizations, and customers, and government departments should actively improve supporting laws and regulations, which can help to promote the transmission efficiency between mediating variables and explanatory variables, and jointly promote the development of green innovation.

# References

1. Aboud, A., & Diab, A. (2018). The impact of social, environmental and corporate governance disclosures on firm value: Evidence from Egypt. Journal of Accounting in Emerging Economies, 8(4), 442–458.
2. Adams, R. B. (2012). Governance and the financial crisis. International Review of Finance, 12(1), 7–38. <https://doi.org/10.1111/j.1468-2443.2011.01147.x>
3. Amel-Zadeh, A., & Serafeim, G. (2018). Why and How Investors Use ESG Information: Evidence from a Global Survey. Financial Analysts Journal, 74(3), 87-103. DOI:10.2469/faj.v74.n3.2
4. Amel-Zadeh, A., & Serafeim, G. (2021). Why and How Investors Use ESG Information: Evidence from a Global Survey. Financial Analysts Journal, 77(3), 87-104. DOI:10.2469/faj.v74.n3.2
5. Amini, M., & Rahmani, A. (2023). Achieving Financial Success by Pursuing Environmental and Social Goals: A Comprehensive Literature Review and Research Agenda for Sustainable Investment. World Information Technology and Engineering Journal, 10, 1286-1293.
6. Anastasiou, D. (2023). Management and Resolution methods of Non-performing loans: A Review of the Literature. Crises and Uncertainty in the Economy, 187-201.
7. Angelini, P,Cetorelli, N.(2003). The effects of regulatory reform on competition in the banking industry. Journal of Money Credit and Banking, 35(5):663-684. <https://doi.org/10.1353/mcb.2003.0033>
8. Aouadi, A., & Marsat, S. (2018). Do ESG controversies matter for firm value? Evidence from international data. Journal of Business Ethics, 151, 1027–1047. https://doi.org/10.1007/s10551-016-3213-8
9. Arena, C., Bozzolan, S., & Michelon, G. (2015). Environmental reporting: Transparency to stakeholders or stakeholder manipulation? An analysis of disclosure tone and the role of the board of directors. Corporate Social Responsibility and Environmental Management, 22(6), 346–361. <https://doi-org.fgul.idm.oclc.org/10.1002/csr.1350>
10. Babic, M., & Sharma, S. E. (2023). Mobilising critical international political economy for the age of climate breakdown. New Political Economy, 1-22.
11. Biggerstaff, L., Blank, B., & Goldie, B. (2019). Do incentives work? Option-based compensation and corporate innovation. Journal of Corporate Finance, 58, 415-430.
12. Boyallian, P., & Ruiz-Verdú, P. (2018). Leverage, CEO risk taking incentives, and bank failure during the 2007-10 financial crisis. Review of Finance, 22(5), 1763–1805. <https://doi.org/10.1093/rof/rfx051>
13. Brunnermeier, SB, Cohen, MA.(2003). Determinants of environmental innovation in US manufacturing industries. Journal of Enviromental Economics and Management, 45(2):278-293. DOI:10.1016/S0095-0696(02)00058-X
14. Cao, C., Tong, X., Chen, Y., et al. (2022). How top management’s environmental awareness affect corporate green competitive advantage: Evidence from China. Kybernetes, 51(3), 1250–1279. <https://doi-org.fgul.idm.oclc.org/10.1108/K-01-2021-0065>
15. Chen, YS,Lai, SB,Wen, CT.(2006).The influence of green innovation performance on corporate advantage in Taiwan. Journal of Business Ethics,67(4): 331-339. DOI:10.1007/s10551-006-9025-5
16. Chen, Y. S., Chang, C. H., & Wu, F. S. (2012). Origins of green innovations: The differences between proactive and reactive green innovations. Management Decision.50:368-398. DOI:10.1108/00251741211216197
17. Chen, Y. S., & Chang, C. H. (2013). The determinants of green product development performance: Green dynamic capabilities, green transformational leadership, and green creativity. Journal of Business Ethics, 116(1), 107-119. DOI:10.1007/s10551-012-1452-x
18. Chen, X., Yi, N., Zhang, L., & Li, D. (2018). Does institutional pressure foster corporate green innovation? Evidence from China's top 100 companies. Journal of cleaner production, 188, 304-311.
19. Chiaramonte, L., Dreassi, A., Girardone, C., & Piserà, S. (2022). Do ESG strategies enhance bank stability during financial turmoil? Evidence from Europe. The European Journal of Finance, 28(12), 1173-1211.
20. Citterio, A., & King, T. (2023). The role of Environmental, Social, and Governance (ESG) in predicting bank financial distress. Finance Research Letters, 51, 103411.
21. Clarkson, P. M., Li, Y., Richardson, G. D., & Vasvari, F. P. (2008). Revisiting the relation between environmental performance and environmental disclosure: An empirical analysis. Accounting, Organizations and Society, 33(4-5), 303-327. DOI:10.1016/j.aos.2007.05.003
22. Conyon, M. J., & He, L. (2012). CEO compensation and corporate governance in China. Corporate Governance: An International Review, 20(6), 575-592. https://doi.org/10.1111/j.1467-8683.2012.00935.x
23. Cui, XG ,Xu, L ,Zhang, HL.(2021). Executive compensation and firm performance: Evidence from cross-listed AH-share firms.International Journal of Finance & Economics,26(1): 88-102. https://doi.org/10.1002/ijfe.1778
24. Dangelico, R. M., & Pujari, D. (2010). Mainstreaming green product innovation: Why and how companies integrate environmental sustainability. Journal of Business Ethics, 95(3), 471-486. DOI:10.1007/s10551-010-0434-0
25. Darnall, N., Henriques, I., & Sadorsky, P. (2008). Do environmental management systems improve business performance in an international setting?. Journal of International Management, 14(4), 364-376. DOI:10.1016/j.intman.2007.09.006
26. DeFries, R., Achard, F., Brown, S., Herold, M., Murdiyarso, D., Schlamadinger, B., & de Souza Jr, C. (2007). Earth observations for estimating greenhouse gas emissions from deforestation in developing countries. Environmental science & policy, 10(4), 385-394.
27. Dietrich, A., & Wanzenried, G. (2011). Determinants of bank profitability before and during the crisis: Evidence from Switzerland. Journal of International Financial Markets, Institutions and Money, 21(3), 307-327. https://doi.org/10.1016/j.intfin.2010.11.002
28. Ding, Z., Li, W., & Zhou, X. (2023). Is the establishment of city commercial banks more efficient in promoting green innovation?. Environmental Science and Pollution Research, 30(16), 47065-47076.
29. Eccles, R. G., & Serafeim, G. (2013). The performance frontier: innovating for a sustainable strategy. Harvard business review, 91(5), 50-60.
30. Eccles, RG,Ioannou, I, Serafeim, G.(2014).The Impact of Corporate Sustainability on Organizational Processes and Performance. Management Science, 60(11): 2835-2857. DOI:10.1287/mnsc.2014.1984.
31. El Ghoul, S ,Guedhami, O , Kwok, CCY,Mishra, DR.(2011). Does corporate social responsibility affect the cost of capital? Journal of Banking & Finance, 35(9): 2388-2406. DOI:10.1016/j.jbankfin.2011.02.007
32. Elzinga, K. G., & Mills, D. E. (2011). The Lerner index of monopoly power: origins and uses. American Economic Review, 101(3), 558-564.
33. Feng, Y., & Ma, X. (2023). The impact of perceived environmental uncertainty on green innovation: evidence from text recognition of Chinese listed companies’ annual reports. Environmental Science and Pollution Research, 1-21.
34. Foos,D,Norden, L,Weber, M.(2010)Loan growth and riskiness of banks. Journal of Banking & Finance, 34(12): 2929-2940. DOI:10.1016/j.jbankfin.2010.06.007
35. Friede, G., Busch, T., & Bassen, A. (2015). ESG and financial performance: aggregated evidence from more than 2000 empirical studies. Journal of Sustainable Finance & Investment, 5(4), 210-233. <https://doi.org/10.1080/20430795.2015.1118917>
36. Ghisetti, C., Mancinelli, S., Mazzanti, M., & Zoli, M. (2017). Financial barriers and environmental innovations: evidence from EU manufacturing firms. Climate Policy, 17(sup1), S131-S147.
37. Ghosh A (2017) Sector-specific analysis of non-performing loans in the US banking system and their macroeconomic impact. J Econ Bus 93:29–45. <https://doi-org.fgul.idm.oclc.org/10.1016/j.jeconbus.2017.06.002>
38. Goss, A ,Roberts, GS.(2011). The impact of corporate social responsibility on the cost of bank loans. Journal of Banking & Finance, 35(7): 1794-1810. DOI:10.1016/j.jbankfin.2010.12.002
39. Green, J. (2023). Comparative capitalisms in the Anthropocene: a research agenda for green transition. New Political Economy, 28(3), 329-346.
40. Hagedoorn, J., & Cloodt, M. (2003). Measuring innovative performance: is there an advantage in using multiple indicators? Research Policy, 32(8), 1365-1379. https://doi.org/10.1016/S0048-7333(02)00137-3
41. Hu, S., Zhang, C., Ji, Y., & Zhang, C. (2023). Does environmental information disclosure tone affect corporate green innovation in China? Based on the institutional perspective. Environment, Development and Sustainability, 1-44.
42. Kahneman D. Attention and effort[M]. Englewood Cliffs, NJ: Prentice-Hall, 1973.
43. Kahneman, D. (2003). Maps of bounded rationality: Psychology for behavioral economics. American economic review, 93(5), 1449-1475.
44. Khan, M, Serafeim, G, Yoon, A. (2016). Corporate sustainability: First evidence on materiality. Accounting Review, 91(6), 1697-1724. DOI:10.2308/accr-51383
45. Kim, K., Choi, S. O., & Lee, S. (2021). The effect of a financial support on firm innovation collaboration and output: Does policy work on the diverse nature of firm innovation?. Journal of the Knowledge Economy, 12, 645-675.
46. Kryzanowski, L., Liu, J., & Zhang, J. (2023). Effect of COVID-19 on non-performing loans in China. Finance Research Letters, 52, 103372.
47. Lerner, A. P. (1934). Economic theory and socialist economy. The Review of Economic Studies, 2(1), 51-61.
48. Liu, S., Jin, J., & Nainar, K. (2023). Does ESG performance reduce banks’ nonperforming loans?. Finance Research Letters, 103859.
49. Long, H., Feng, G. F., Gong, Q., & Chang, C. P. (2023). ESG performance and green innovation: An investigation based on quantile regression. Business Strategy and the Environment, 32(7), 5102-5118.
50. Louzis DP, Vouldis AT, Metaxas VL (2012) Macroeconomic and bank-specific determinants of non-performing loans in Greece: A comparative study of mortgage, business and consumer loan portfolios. J Bank Finance 36:1012–1027. https://doi-org.fgul.idm.oclc.org/10.1016/j.jbankfin.2011.10.012
51. Luo, J., Wang, H., Raithel, S., & Zheng, Q. (2015). Corporate social performance, analyst stock recommendations, and firm future returns. Strategic Management Journal, 36(1), 123-136. DOI:10.1002/smj.2219
52. MacKinnon, DP,Lamp, SJ.(2021). A Unification of Mediator, Confounder, and Collider Effects. Prevention Science,22(8): 1185-1193. <https://doi.org/10.1007/s11121-021-01268-x>
53. Manz, F. (2019). Determinants of non-performing loans: What do we know? A systematic review and avenues for future research. Management review quarterly, 69(4), 351-389.
54. Nevrela, A. (2022). Association between corporate social responsibility and goodwill impairment: evidence from the European Union. International Journal of Economics and Accounting, 11(1), 42-72.
55. Pankratov, E. L., & Trifonov, Y. V. (2021). On analysis of the competitiveness of industrial enterprises. Journal of the Knowledge Economy, 1-8.
56. Peng, L., & Xiong, W. (2006). Investor attention, overconfidence and category learning. Journal of Financial Economics, 80(3), 563-602.
57. Rabaya, A. J., & Saleh, N. M. (2022). The moderating effect of IR framework adoption on the relationship between environmental, social, and governance (ESG) disclosure and a firm's competitive advantage. Environment, Development and Sustainability, 24(2), 2037-2055.
58. Rajesh, R.(2020). Exploring the sustainability performances of firms using environmental, social, and governance scores. Journal of Cleaner Production,247: 119600. DOI:10.1016/j.jclepro.2019.119600
59. Rehman, S. U., Kraus, S., Shah, S. A., Khanin, D., & Mahto, R. V. (2021). Analyzing the relationship between green innovation and environmental performance in large manufacturing firms. Technological Forecasting and Social Change, 163, 120481.
60. Schaltegger, S., & Wagner, M. (2011). Sustainable entrepreneurship and sustainability innovation: categories and interactions. Business Strategy and the Environment, 20(4), 222-237. DOI:10.1002/bse.682
61. Servaes, H., & Tamayo, A. (2013). The impact of corporate social responsibility on firm value: The role of customer awareness. Management science, 59(5), 1045-1061. DOI:10.1287/mnsc.1120.1630
62. Silva, F., & Carreira, C. (2012). Do financial constraints threat the innovation process? Evidence from Portuguese firms. Economics of Innovation and New Technology, 21(8), 701-736.
63. Singh, SK,Del Giudice, M ,et.al.(2020). Green innovation and environmental performance: The role of green transformational leadership and green human resource management. Technological Forecasting and Social Change, 150: 119762. <https://doi.org/10.1016/j.techfore.2019.119762>
64. Takalo, S. K., & Tooranloo, H. S. (2021). Green innovation: A systematic literature review. Journal of Cleaner Production, 279, 122474.
65. Triguero, Á., Moreno-Mondéjar, L., & Davia, M. A. (2013). Drivers of different types of eco-innovation in European SMEs. Ecological Economics, 92, 25-33. DOI:10.1016/j.ecolecon.2013.04.009
66. Wang, J., Ma, M., Dong, T., & Zhang, Z. (2023). Do ESG ratings promote corporate green innovation? A quasi-natural experiment based on SynTao Green Finance's ESG ratings. International Review of Financial Analysis, 87, 102623.
67. Wu, S., & Li, Y. (2023). A Study on the Impact of Digital Transformation on Corporate ESG Performance: The Mediating Role of Green Innovation. Sustainability, 15(8), 6568.
68. Xie, XM ,Huo, JG, Zou, HL .(2019). Green process innovation, green product innovation, and corporate financial performance: A content analysis method. Journal of Business Research,101: 697-706. <https://doi.org/10.1016/j.jbusres.2019.01.010>
69. Xia, Y., & Liu, P. (2022). Does bank competition promote corporate green innovation? Evidence from the location of bank branches. China & World Economy, 30(2), 84-116.
70. Xu, L., Zhang, X., & Zhao, J. (2023). Limited investor attention and biased reactions to information: Evidence from the COVID-19 pandemic. Journal of Financial Markets, 62, 100757.
71. Yin, XN , Li, JP , Su, CW.(2023). How does ESG performance affect stock returns? Empirical evidence from listed companies in China. Heliyon,9:5. https://doi.org/10.1016/j.heliyon.2023.e16320
72. Zalewska, A. (2023). Bankers' remuneration reforms and new challenges. Corporate Governance: An International Review, 31(1), 161-177.
73. Zhang, B,Yang, Y , Bi, J .(2011).Tracking the implementation of green credit policy in China: Top-down perspective and bottom-up reform. Journal of Enviromental Management, 92(4): 1321-1327. DOI:10.1016/j.jenvman.2010.12.019
74. Zhang, C., & Chen, D. (2023). Do environmental, social, and governance scores improve green innovation? Empirical evidence from Chinese-listed companies. Plos one, 18(5), e0279220.
75. Zhao, J., Chankoson, T., Cheng, W., & Pongtornkulpanich, A. (2023). Executive compensation incentives, innovation openness and green innovation: evidence from China's heavily polluting enterprises. European Journal of Innovation Management.
76. Zhao, Q., Li, X., & Li, S. (2023). Analyzing the Relationship between Digital Transformation Strategy and ESG Performance in Large Manufacturing Enterprises: The Mediating Role of Green Innovation. Sustainability, 15(13), 9998.
77. Zhou, B., Li, Y. M., Sun, F. C., & Zhou, Z. G. (2021). Executive compensation incentives, risk level and corporate innovation. Emerging Markets Review, 47, 100798.
78. Zhu, S., Sun, H., Zhang, B., Yang, Z., & Xia, X. (2023). Bilateral Effects of ESG Responsibility Fulfillment of Industrial Companies on Green Innovation. Sustainability, 15(13), 9916.

# Competing interests

We acknowledge financial support from the Social Science Foundation of Qinghai Province (Grant No. 22Y013) and the Social Science Foundation of Jilin Province (Grant No. 2021B56). The authors declare that they have no relevant or material financial interests that relate to the research described in this paper.

# Data availability

The datasets generated during and/or analysed during the current study are available from the corresponding author on reasonable request.

# Ethical approval

This article does not contain any studies with human participants performed by any of the authors.

# Informed consent

This article does not contain any studies with human participants performed by any of the authors.
